# Case Report: Multiple immune related adverse events in a patient with metastatic melanoma

**DOI:** 10.3389/fimmu.2025.1677544

**Published:** 2025-12-16

**Authors:** Neilmegh L. Varada, Olivia Yang, David J. Savage

**Affiliations:** Department of Internal Medicine, University of New Mexico Comprehensive Cancer Center, Albuquerque, NM, United States

**Keywords:** immune-related adverse events, melanoma, immune checkpoint inhibitors, nivolumab, myocarditis, hepatitis, relatlimab, combination immunotherapy

## Abstract

**Introduction:**

Immune related adverse events (irAEs) are a well-recognized potential complication of immunotherapy treatment. Immunotherapy works at the level of T-cells and tumors to blunt checkpoints that normally suppress overactivation of the immune response. While this leads to a therapeutic benefit in many cases, the dysregulated immune system can also attack healthy parts of the body, leading to toxicity. For stage IV melanoma, combination checkpoint inhibition with multiple drugs agents is the preferred frontline treatment, however, this can increase the risk of irAEs. This case describes a person treated with the Lymphocyte-activation gene 3 (LAG-3) inhibitor relatlimab and the Programmed cell death protein 1 (PD-1) inhibitor nivolumab for stage IV melanoma who subsequently developed four distinct and significant toxicities.

**Case description:**

An 80-year-old male with a history of melanoma was diagnosed with stage IV melanoma. He was started on treatment with relatlimab/nivolumab. One month later he began to experience Liver Function Test (LFT) elevations that were < 2x upper limit of normal (ULN). After Cycle 2, labs showed worsening of transaminitis, this time nearly 2x ULN. He started on a steroid taper, returning his LFTs to normal, and he was treated with Cycle 3. In the next month, LFTs worsened to >3x ULN, he developed a rash, and the patient developed primary hypothyroidism. Treatment was discontinued and he started thyroid hormone replacement. One month after cycle 3 was given, he was admitted with an acute heart failure exacerbation secondary to myocarditis. Multiple attempts to taper steroids were made, but LFTs worsened each time. He was ultimately started on mycophenolate mofetil in three months later and tapered off steroids completely. His LFTs, rash, and myocarditis resolved. Patient remains on active surveillance with permanent discontinuation of immunotherapy.

**Discussion:**

This patient developed grade 2 primary hypothyroidism, grade 3 myocarditis, grade 2 dermatitis, and grade 3 hepatitis from three treatments with relatlimab/nivolumab. There were some early indications of LFT changes prior to cycle 3, but symptoms were most appreciable after cycle 3. The relatlimab/nivolumab combination was selected because of the lower incidence of irAEs reported in Relativity-047 compared to the CheckMate trials with ipilimumab/nivolumab. This case demonstrates the challenges of managing multiple irAEs in a single patient. It also is an acute reminder that immune activation can take time, and that irAEs can persist for a long period of time or indefinitely after treatment is discontinued.

**Conclusions:**

irAEs occur in up to 30% of patients who receive checkpoint immunotherapy for stage IV melanoma treatment. It is possible to have multiple toxicities in a single patient. Management requires prolonged immune suppression, correction of endocrine abnormalities, and coordination with a multidisciplinary team. Physicians should have a low threshold to pause treatment at early laboratory signs of an evolving irAE.

## 1 Introduction

Immune checkpoint inhibitors (ICIs) have revolutionized the treatment landscape for metastatic melanoma, leveraging the immune system’s ability to recognize and attack tumor cells (1). The combination of PD-1 and Cytotoxic T-lymphocyte associated protein 4 (CTLA-4) inhibitors, though effective (2), has been associated with high rates of immune-related adverse events (irAEs). These occur when the activated immune system has off target effects on non-cancerous tissues in the body. In 2022, the U.S. Food and Drug Administration approved a novel dual immunotherapy of relatlimab, a lymphocyte activation gene-3 (LAG-3) inhibitor in combination with nivolumab for patients with unresectable or metastatic melanoma. This approval was based on the phase III RELATIVITY-047 trial, which demonstrated improved progression-free survival and a more favorable safety profile compared to nivolumab plus ipilimumab (3).

Although the incidence of high-grade irAEs with relatlimab/nivolumab appears lower than with earlier dual ICI regimens, clinically significant toxicities remain a concern. The most reported irAEs involve the skin, gastrointestinal tract, liver, and endocrine organs, and they can occur even late in the treatment course or persist long-term (4–6). Myocarditis, while rare, remains one of the most serious and potentially fatal irAEs (7).

We present a unique case of a patient treated with relatlimab/nivolumab who developed four distinct irAEs, specifically hepatitis, dermatitis, thyroiditis, and myocarditis, illustrating the potential for multisystem immune activation even with newer dual immunotherapy regimens. This case highlights the need for continued vigilance and multidisciplinary management in patients receiving immune checkpoint inhibitors.

## 2 Case description

**Figure 1:**
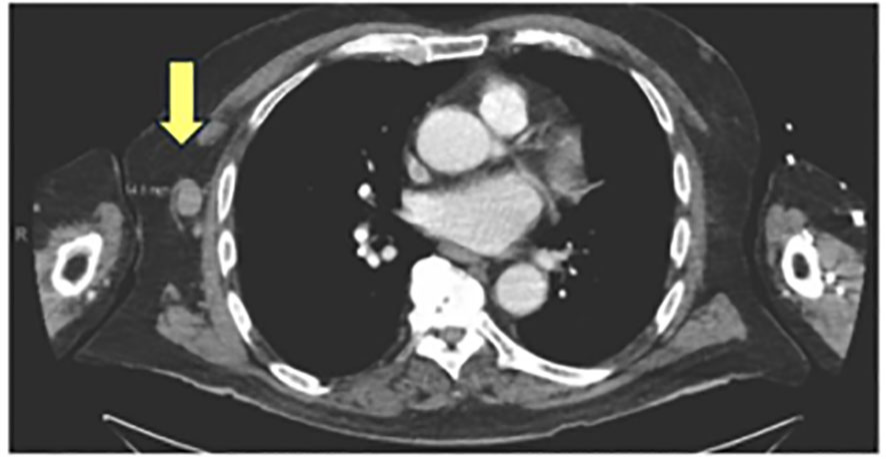
Right axillary lymphadenopathy that was biopsy proven to be stage IV melanoma.

**Figure 2:**
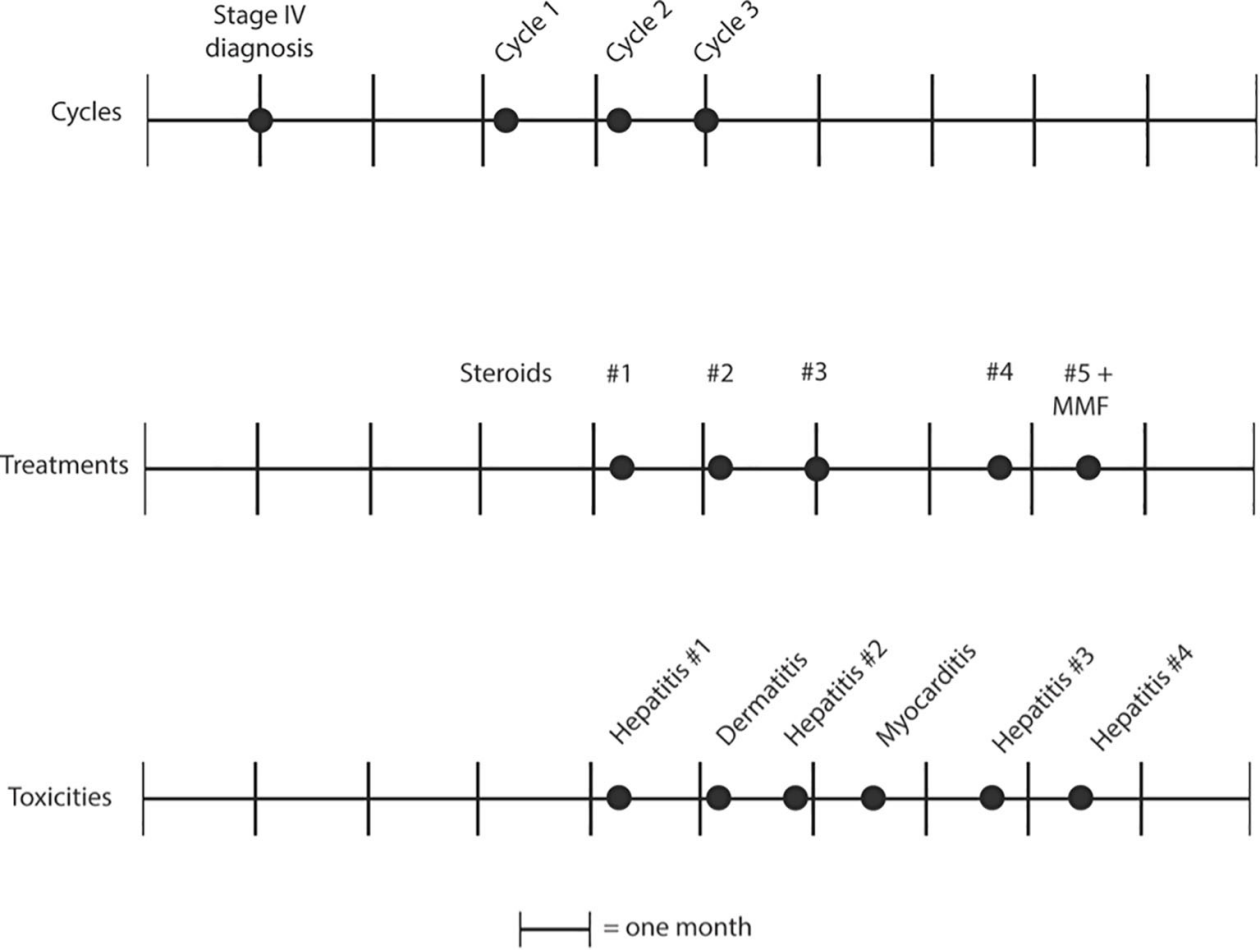
Timeline of treatments and toxicities for the patient.

**Figure 3:**
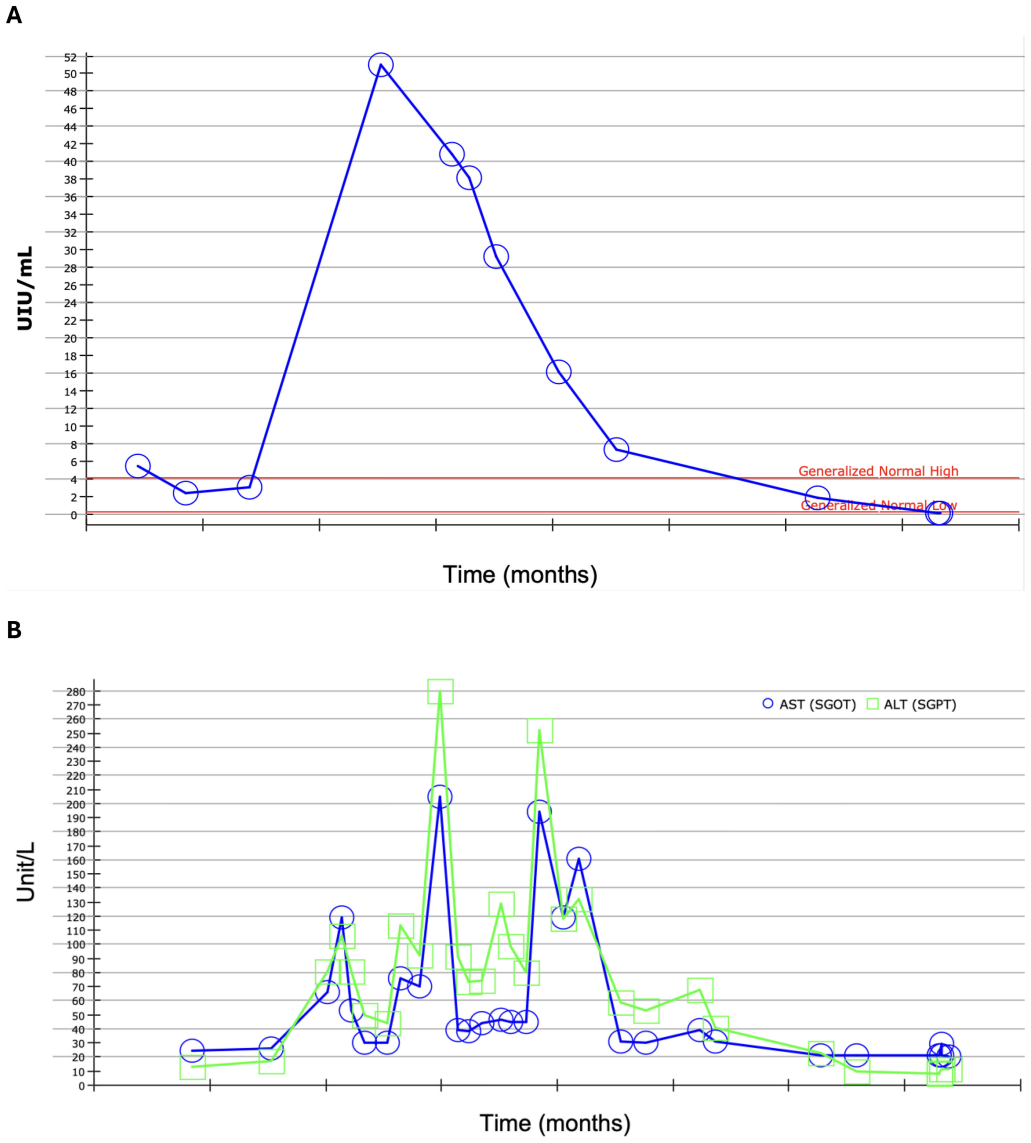
**(A)** TSH trend demonstrating primary hypothyroidism and **(B)** Aspartate Aminotransferase (AST, blue) and Alanine Aminotransferase (ALT, green) levels reflect the hepatitis trend over time for this patient.

**Figure 4:**
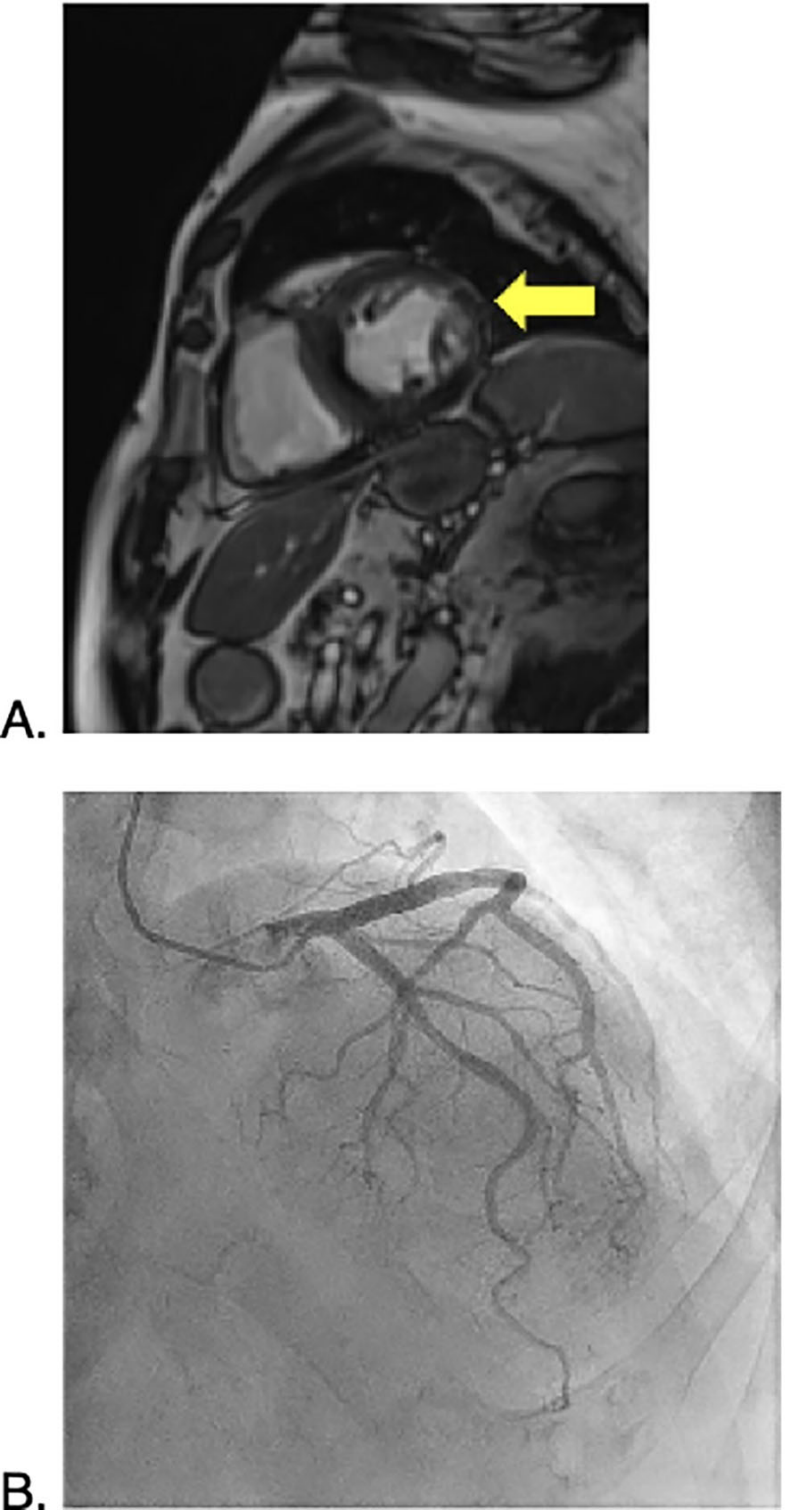
Cardiac MRI **(A)** showing mild edema signal in the left ventricular mid lateral wall with early and delayed moderate enhancement consistent with myocarditis, and coronary angiography **(B)** showing mild atherosclerosis.

The patient is an 80-year-old male with a history of melanoma *in situ* on the right forehead, Gastroesophageal reflux disease (GERD), Benign Prostate Hyperplasia (BPH) and ischemic and hemorrhagic Cerebrovascular accident (CVA), who presented to the emergency department for with slurred speech. He received an extensive workup for a stroke. During this work up, a Computed tomography (CT) angiogram of the neck showed diffuse enlarged cervical lymphadenopathy (**Figure 1**). On account of this, the patient underwent whole-body CT imaging given his history of melanoma and was found to have diffuse axillary and cervical lymphadenopathy. An interventional radiology (IR) guided biopsy was performed a month later, showing metastatic melanoma and a right axillary lymph node. The patient was referred to a medical oncologist to discuss treatment options. The patient had an ECOG performance status of 1. He lived by himself doing all of his ADL’s independently, but did get assistance from his daughter for certain tasks. Given his overall performance status, he started treatment with Cycle 1 of relatlimab/nivolumab (Opdualag) 2 months after the biopsy (**Figure 2**). When he returned for Cycle 2 a month later, it was noted that he was already starting to experience early signs of transaminitis (Alanine transaminase (ALT) 81, Aspartate transaminase (AST) 66 (**Figure 3A**). This was considered to a grade 1irAE as this was less than three times the upper limit of normal, as such he was treated with Cycle 2. Shortly thereafter he began to experience whole body pruritus without rash, which was treated with diphenhydramine. He also experienced oral mucosal pain without sores or mucosal breakdown. These were characterized as grade 2 pruritus and grade 2 oral mucosal inflammation. Labs were checked during this cycle showed further worsening of this transaminitis (ALT 106, AST 119). Due to this, he started on high-dose steroids with a quick taper as he now fell in a grade 2 irAE. Within a few weeks his LFTs returned to a normal range, and he received Cycle 3 treatment. Within one month, he had a rapid worsening of his LFTs, which peaked one month later (ALT 208, AST 280), and was diagnosed with a grade 3 irAE. He also had apparent signs of primary hypothyroidism at that time, with a Thyroid-stimulating hormone (TSH) of 51 (**Figure 3B**). The decision was made to permanently discontinue treatment. He started on levothyroxine, and he was again started on weight-based steroids with a long taper over 4 weeks. His LFTs returned to near normal but then the taper, but discontinuation of steroids led to worsening of LFTs. In addition, near the end of the taper, the patient was admitted to the hospital for an episode of acute decompensated heart failure. A cardiac Magnetic resonance imaging (MRI) performed at that time showed evidence of myocarditis, and the patient had an elevated serum troponin (**Figure 4**). He was discharged and continued 60 mg daily prednisone with a plan for a slow taper while plans were made to have the patient seen in the hepatology clinic. Testing for autoimmune and viral etiologies of hepatitis was performed and all testing was negative. Patient underwent a liver biopsy that showed mild to moderate portal hepatitis and mild portal fibrosis. The patient was seen in the GI liver clinic 1 month later and was started on mycophenolate mofetil (MMF) 500 mg in parallel with a slow prednisone taper. LFTs were still elevated at that time (ALT 132, AST 161). Since starting MMF, the patient’s LFTs have returned to normal range. His TSH is now in the normal range; his heart failure symptoms are well-managed.

## 3 Discussion

This case illustrates the complexity and unpredictability of immune-related adverse events (irAEs) in patients receiving dual checkpoint inhibition with relatlimab and nivolumab. While this combination has demonstrated a favorable safety profile relative to ipilimumab/nivolumab in the RELATIVITY-047 trial (3), our patient experienced four distinct and clinically significant irAEs: hepatitis, dermatitis, thyroiditis, and myocarditis. These occurred at different points in the treatment timeline and persisted after discontinuation of therapy, reinforcing the need for long-term monitoring and multidisciplinary care.

One strength of this case was the early recognition of evolving laboratory abnormalities, which prompted timely discontinuation of immunotherapy. Although initial symptoms were subtle, the decision to pause treatment allowed for further evaluation and treatment. Nevertheless, the patient still progressed to develop grade 3 myocarditis and hepatitis, underscoring the limitations of current surveillance strategies and treatments. The variable onset of irAEs in this patient, from early and persistent hepatic transaminitis to delayed-onset myocarditis with an acute heart failure exacerbation, reflects the heterogeneity of immune activation and the need for sustained vigilance.

The literature suggests that irAEs affect 15–90% of patients receiving immunotherapy, depending on the agent and definitions used (4, 6). In PD-1 inhibitor monotherapy, the incidence of any-grade irAEs is approximately 30% (5). In the RELATIVITY-047 trial, although early grade 3–4 toxicities were rare during the first 8 weeks, nearly 26% of patients developed them later, and one-third discontinued treatment due to toxicity (3). Adrenal insufficiency was the most common severe irAE reported (8). While the incidence of toxicity may be lower than with ipilimumab-containing regimens, the potential for multiple, severe, and prolonged irAEs remains clinically significant.

Importantly, our case highlights several key considerations for clinical practice:

### 3.1 Early detection and action

Physicians should maintain a high index of suspicion for irAEs, even in patients on regimens perceived as lower risk. Laboratory abnormalities or vague symptoms should prompt early investigation and, if necessary, withholding of further immunotherapy (5).

### 3.2 Escalation of immunosuppression

While corticosteroids remain first-line therapy for most irAEs (9), escalation to second-line immunosuppressants—such as mycophenolate mofetil, infliximab, tocilizumab, or vedolizumab—may be necessary to control severe or refractory events, as was the case in our patient (10).

### 3.3 Multispecialty coordination

Effective management of multisystem irAEs often requires input from several specialists. Our patient benefited from collaborative care involving cardiology, endocrinology, and gastroenterology. Such coordination may be challenging in community or rural settings where access to subspecialists is limited.

### 3.4 Protracted and late-onset toxicity

Unlike traditional chemotherapy, immunotherapy-related toxicity may occur months after treatment initiation—or even after treatment cessation—as seen in this patient’s delayed-onset myocarditis. This has implications for survivorship care and long-term follow-up protocols.

The pathophysiology of irAEs remains incompletely understood. Current theories are based on current understanding of immune checkpoint pathways in autoimmune diseases. Proposed mechanisms include autoreactive T-cell activation, cross-reactivity with self-antigens, and cytokine-mediated inflammation (6, 11–13). Some evidence suggests that patients with multiple irAEs may experience improved oncologic outcomes (14, 15). Our patient did have improvement in disease with resolution of pathologic adenopathy, but it is too early to know whether this will be a sustained response. Further research is needed to clarify predictive biomarkers and optimal management strategies for patients with complex immunotherapy toxicity. Given the low burden of disease for this patient, a biomarker indicating a high risk of an irAE may have led us to postpone all treatment entirely.

## 4 Conclusion

As immunotherapy becomes increasingly central in the treatment of advanced melanoma, clinicians must be prepared to recognize and manage its immune-related complications. This case underscores that multiple severe irAEs can occur in a single patient, even with newer regimens thought to have a lower toxicity burden. Prompt identification, appropriate grading, escalation of immunosuppression when needed, and multidisciplinary care are all critical to optimizing outcomes. Physicians should maintain a low threshold to pause treatment in the setting of early signs of irAEs and ensure long-term follow-up for delayed or persistent immune toxicities.

## Data Availability

The original contributions presented in the study are included in the article/supplementary material. Further inquiries can be directed to the corresponding author.
